# New challenges for efficient usage of *Sargassum fusiforme* for ruminant production

**DOI:** 10.1038/s41598-020-76700-3

**Published:** 2020-11-12

**Authors:** You Young Choi, Shin Ja Lee, Ye Jun Lee, Hyun Sang Kim, Jun Sik Eom, Sam Churl Kim, Eun Tae Kim, Sung Sill Lee

**Affiliations:** 1 Division of Applied Life Science (BK21 Plus) and Institute of Agriculture and Life Science (IALS), Gyeongsang National University, Jinju, 52828 South Korea; 2 Institute of Agriculture and Life Science and University-Centered Labs, Gyeongsang National University, Jinju, 52828 South Korea; 3 National Institute of Animal Science, Rural Development Administration (RDA), Cheonan, 31000 South Korea

**Keywords:** Plant sciences, Environmental sciences

## Abstract

*Sargassum fusiforme*, which is a type of brown algae, can provide fiber and minerals to ruminant diets. In this study, dried *S. fusiforme* was tested in vitro at four different doses 1, 3, 5, and 10% of the total ration for its effect on ruminal fermentation characteristics, and gas profiles when incubated for 72 h. At a level of 1 and 10%, *S. fusiforme* supplementation augmented total volatile fatty acid (VFA) concentrations compared to that with 0% supplementation. In addition, total gas, methane, and carbon dioxide emissions significantly decreased at 3 and 24 h of incubation at this dose. An in situ trial was performed for 72 h with *S. fusiforme* to evaluate it as a potential feed ingredient by comparing its degradation parameters with timothy hay (*Phleum pretense*). ^1^H nuclear magnetic resonance spectroscopy profiling was used to identify and quantify metabolites of *S. fusiforme*. Mannitol, guanidoacetate and ethylene glycol were largely accumulated in *S. fusiforme*. Moreover, nutritious minerals for feed ingredients were present in *S. fusiforme*. Whereas a high concentration of arsenic was found in *S. fusiforme*, it was within the allowable limit for ruminants. Our results suggest that *S. fusiforme* could represent an alternative, renewable feed ingredient for ruminant diets, with nutritional, as well as environmental, benefits.

## Introduction

Concomitant with the growing interest in algae is the need to identify new, bioactive compounds, potential biofuel developments and potential animal feed sources^1–4^. Feed cost is a major concern for the livestock industry because it constitutes up to 50% of the total production cost in Korea. In 2018, the National Institute of Fisheries Science in Korea reported that total *Sargassum fusiforme* (*S. fusiforme*; Tot, Hijiki) production was approximately 54,624 tons, with residues from the production process such as harvesting, processing, and selling, accounting for 5% of total production. Presently, most algal residues are dumped into the ocean, which might adversely affect the marine environment if it exceeds the self-cleaning capacity of the subsea ecosystem. As countries such as Korea do not have vast agricultural areas for forage cultivation, the utilization of algal and its residues as an alternative feed supplement should be considered. This could simultaneously reduce animal feed costs while considering the potential relationship with environmental pollution.

Algae are fast growing organisms that contain high levels of minerals, carbohydrates, and protein. Among these, carbohydrates are the most abundant constituent, accounting for 60 -70% of total algal content in the form of cellulose and starch^5^. Previous studies have reported that algae-derived bioactive molecules such as polysaccharides, phlorotannins, minerals, and omega 3 fatty acids play an important role as prebiotics, and could confer antimicrobial, antioxidant, and immunomodulatory benefits to their users as well^6^. *S. fusiforme*, a type of brown algae, is edible and contains many different types of polysaccharides, such as anionic sulfated polysaccharides (fucoidan), carboxylated polysaccharide (alginate), laminarin, and mannitol, which exert positive biological effects. *S. fusiforme*-derived nutrients could be used to enhance growth performance in ruminants and rumen fermentation characteristics because they are rich in carbohydrates, readily available minerals, and vitamins and do not contain anti-nutritional factors^7^.

Ruminants have an intricate microbial ecosystem in the rumen (which resembles the environment in a bioreactor), where most of their energy in the form of volatile fatty acids (VFAs) is produced; additionally, methane is produced during microbial fermentation^8^. Unlike many research areas that consider methane a new energy source, the field of ruminant nutrition aims to improve fermentation efficiency by reducing enteric methane production and preventing the accumulation of reduced equivalents in the rumen. Decreasing enteric methane production from ruminants can prevent up to 15% energy loss^9^. Recently, many studies have suggested strategies to reduce methane emission from ruminants using algae^2,10^. Based on in vitro and in situ studies, we hypothesized that such applications would reveal the extent to which algal can be used as a ruminant feed while mitigating methane emissions and improving productivity. Thus, the objectives of the current study were to (1) investigate the physicochemical and nutritional profiles of *S. fusiforme*, (2) determine degradation parameters (how nutrients digested in sections of the digestive tract can be absorbed and be available to ruminants), and (3) manipulate gas emission using *S. fusiforme*.

## Results

### Chemical composition of S. fusiforme

**Figure 1:**
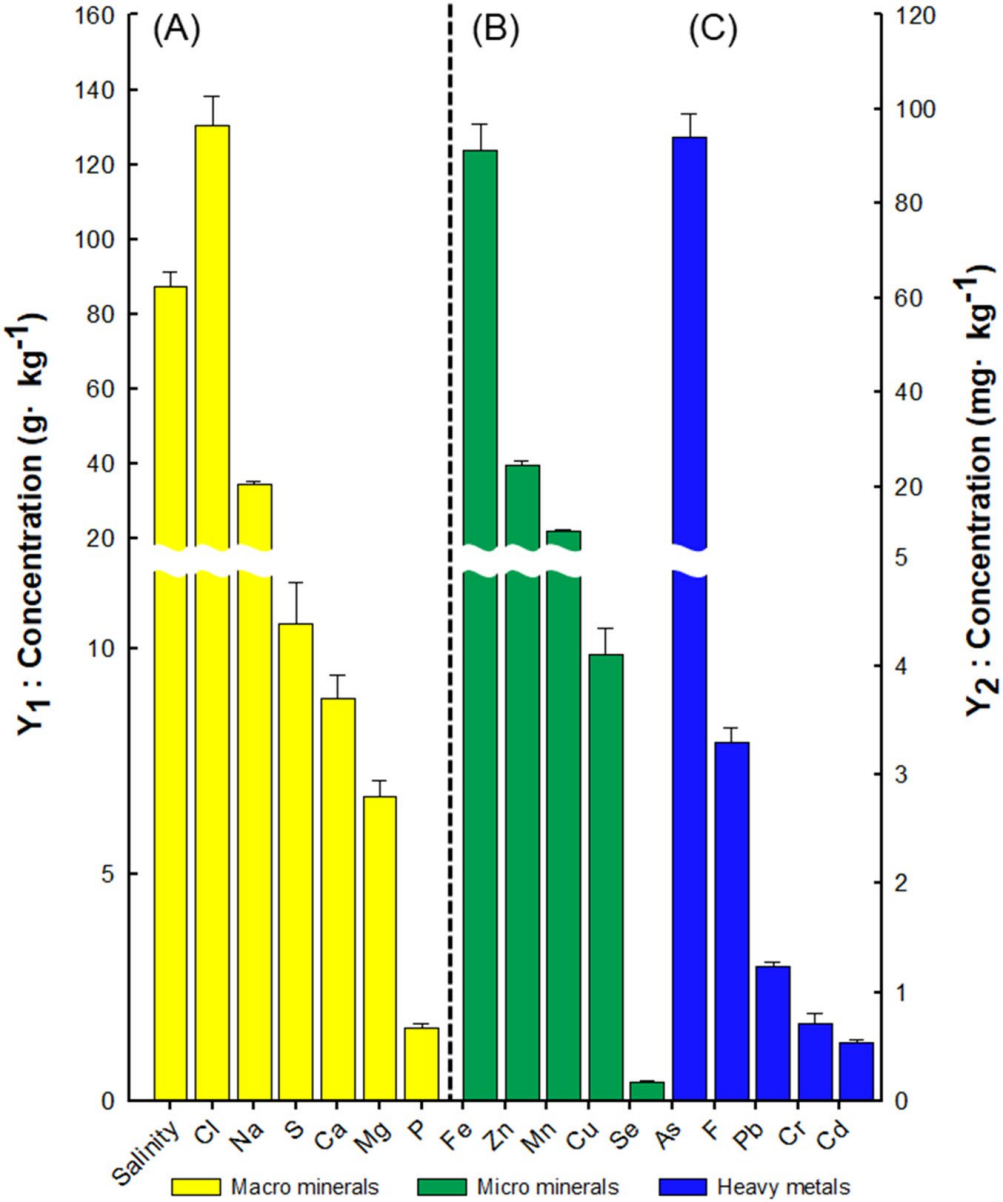
(**A**) macro mineral, (**B**) micro mineral and (**C**) heavy metal concentrations of *Sargassum fusiforme*. Y_1_ axis represents the concentrations of (**A**) macro minerals and Y_2_ axis represent (**B**) micro minerals and (**C**) heavy metals concentrations. Error bars indicate the standard error of the mean (n = 3). *Co and Hg were not detected.

Compared to timothy hay, brown algae *S. fusiforme* had a typically high moisture content 870.6 ± 7.44 g kg^−1^ as -raw, 74.8 ± 0.75 g∙kg^−1^ dry matter (DM) and contains minerals crude ash (CA), 416.2 ± 15.68 g∙kg^−1^ DM, nitrogen free extract (NFE) 430.1 ± 18.48 g∙kg^−1^ DM, and non-fiber carbohydrate (NFC) 291.0 ± 13.37 g∙kg^−1^ DM, while has a relatively low proportion of neutral detergent fiber (NDF), 195.8 ± 1.86 g∙kg^−1^ and acid detergent fiber (ADF), 130.3 ± 4.84 g∙kg^−1^ and gross energy 10.6 ± 0.12 MJ∙kg^−1^ DM (Table 1). Mineral and heavy metal concentrations are shown in Fig. 1. Macro minerals with higher concentrations were salinity 87.4 ± 3.94 g∙kg^−1^ DM, chloride 130.05 ± 7.69 g∙kg^−1^ DM, and sodium 34.40 ± 0.90 g∙kg^−1^ DM. Micro minerals consisted of iron 91.41 ± 5.42 mg∙kg^−1^ DM, zinc 24.53 ± 0.87 mg∙kg^−1^ DM, and manganese 10.66 ± 0.30 mg∙kg^−1^ DM. The heavy metal with the highest content found in *S. fusiforme* was arsenic 94.17 ± 4.96 mg∙kg^−1^ DM, whereas that with the lowest content was cadmium 0.53 ± 0.04 mg∙kg^−1^ DM.

**Table 1:** Chemical compositions of timothy hay and *Sargassum fusiforme.*

Ingredients	Timothy hay	*Sargassum fusiforme*
**Chemical composition (DM, g∙kg**^**−1**^**)**		
Moisture (as-raw basis)	–	870.6 ± 7.44
Moisture (as-DM basis)	122.0 ± 5.45	74.8 ± 0.75
CP	94.0 ± 1.53	90.7 ± 1.47
EE	19.0 ± 0.50	6.3 ± 0.68
CF	356.0 ± 4.74	56.7 ± 3.69
CA	85.0 ± 0.74	416.2 ± 15.68
Ca	2.1 ± 0.01	8.9 ± 0.50
P	1.1 ± 0.01	1.6 ± 0.11
NFE	446.0 ± 6.88	430.1 ± 18.48
**Cell wall constituent (DM, g∙kg**^**−1**^**)**		
NDF	531.8 ± 2.13	195.8 ± 1.86
ADF	305.7 ± 1.17	130.3 ± 4.84
NFC	270.2 ± 4.54	291.0 ± 13.37
Lignin	48.0 ± 0.78	100.1 ± 2.70
Gross Energy (DM, MJ∙kg^−1^)	18.5 ± 0.16	10.6 ± 0.12

ADF, acid detergent fiber; Ca, calcium; CA, crude ash; CF, crude fiber; CP, crude protein; DM, dry matter; EE, ether extract; NDF, neutral detergent fiber; NFC, non-fiber carbohydrate; NFE, nitrogen free extract; P, phosphorus.

All values represent the mean of triplicates ± standard deviation.

### In vitro ruminal fermentation characteristics

The dose–response of ruminal fermentation characteristics to supplementation with *S. fusiforme* is shown in Table 2. All pH values obtained in this study were within the normal range of 5.65 to 7.24, and no significant differences (*P* > 0.05) observed across the fermentation periods. At 24 h incubation, DM digestibility decreased linearly (linear effect: *P* = 0.0027) with 1 and 10% *S. fusiforme* supplementation, showing a minimum DM digestibility compared to that with 0% supplementation, but this rapidly increased after 48 h incubation. Likewise, the microbial growth rate at 9 h incubation demonstrated a linear response (linear effect: *P* = 0.0360), such that 1% *S. fusiforme* supplementation resulted in higher values compared to those with 0% supplementation, but values were intermediate and similar after 12 h incubation.

**Table 2:** Dose response of *Sargassum fusiforme* on in vitro ruminal fermentation characteristics.

Parameters	*Sargassum fusiforme*					SEM	P value		
	0%	1%	3%	5%	10%		*T*	*L*	*Q*
**pH**									
3 h	7.12	7.23	7.12	7.24	7.11	0.04	0.1061	0.5200	0.1423
6 h	7.06	7.02	6.98	7.04	7.03	0.06	0.8450	0.9530	0.6015
9 h	6.88	6.72	6.84	6.94	6.84	0.07	0.2749	0.5613	0.4545
12 h	6.84	6.76	6.66	6.62	6.80	0.07	0.2678	0.8500	0.0337
24 h	6.03	6.42	6.04	6.19	6.33	0.10	0.0548	0.2426	0.5186
48 h	6.01	5.85	5.92	5.87	6.02	0.06	0.2866	0.3879	0.1205
72 h	5.67	5.68	5.70	5.71	5.65	0.09	0.9905	0.8256	0.6547
**DM digestibility (g∙kg**^**−1**^**)**									
3 h	166.9^b^	173.8^b^	195.6^ab^	189.6^ab^	204.1^a^	6.23	0.0093	0.0014	0.1541
6 h	211.6	216.1	200.3	220.1	221.5	5.50	0.1191	0.1480	0.4605
9 h	216.4	246.1	229.7	219.6	240.6	13.23	0.4767	0.5802	0.6640
12 h	234.3	252.1	265.4	284.1	264.2	17.16	0.3900	0.2385	0.1211
24 h	397.7^a^	312.4^b^	413.2^a^	392.3^a^	335.8^b^	11.12	0.0027	0.0914	0.0135
48 h	413.9	406.7	399.7	397.9	376.1	11.13	0.2651	0.0362	0.9415
72 h	489.8	484.6	471.7	484.0	473.9	12.82	0.8337	0.4706	0.7665
**Microbial growth rate (OD at 550 nm)**									
3 h	0.38	0.38	0.41	0.38	0.39	0.02	0.9064	0.7993	0.8297
6 h	0.37	0.37	0.35	0.38	0.40	0.02	0.3739	0.1143	0.6273
9 h	0.48^b^	0.57^a^	0.49^ab^	0.50^ab^	0.53^ab^	0.02	0.0360	0.5337	0.5223
12 h	0.37	0.40	0.44	0.42	0.42	0.03	0.5549	0.3865	0.2488
24 h	0.43	0.38	0.51	0.46	0.43	0.03	0.1932	0.6408	0.1216
48 h	0.40	0.42	0.46	0.44	0.40	0.03	0.5453	0.7845	0.1262
72 h	0.38	0.34	0.38	0.35	0.33	0.03	0.7302	0.4491	0.9460

SEM, standard error of the mean, n = 3; DM, dry matter; OD, optical density; T, treatment; L, linear; Q, quadratic.

^a,b^Means in a row followed by different superscript letters are significantly different (*P* < 0.05).

**Figure 2:**
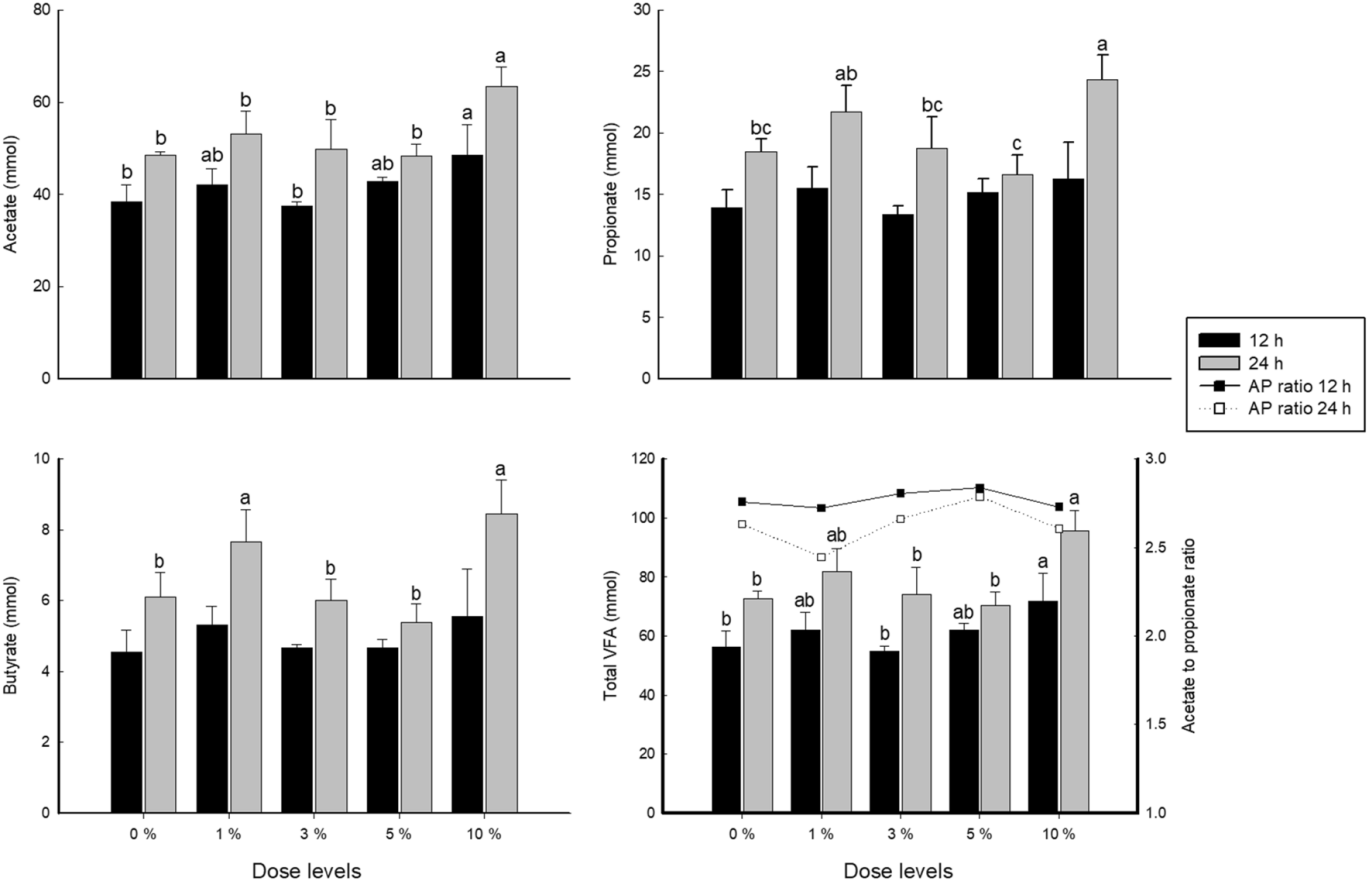
Dose–response of *Sargassum fusiforme* on VFA production 12 and 24 h after in vitro incubation. Error bars indicate the standard error of the mean (n = 3). AP ratio means acetate to propionate ratio. ^a,b,c^Different superscript letters indicate a significant difference (*P* < 0.05).

As indicated in Fig. 2, the inclusion of *S. fusiforme* at 1 and 10% increased total VFA concentrations (linear effect: *P* = 0.0089, *P* = 0.0267, respectively), acetate concentrations (linear effect: *P* = 0.0085, *P* = 0.0189), propionate concentrations (linear effect: *P* = 0.1683, quadratic effect: *P* = 0.0169) and butyrate concentrations (linear effect: *P* = 0.2373, quadratic effect: *P* = 0.0046) more than those with 0% inclusion after 12 and 24 h of incubation.

**Figure 3:**
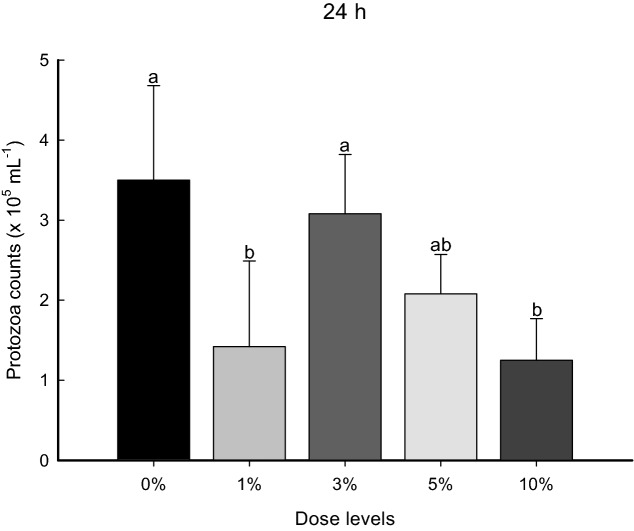
Dose–response of *Sargassum fusiforme* on protozoal numbers (× 10^5^ mL^−1^) after 24 h of in vitro incubation. Error bars indicate standard error of the mean (n = 3). ^a,b^Different superscript letters indicate a significant difference (*P* < 0.05).

The number of rumen protozoa at 24 h decreased linearly (linear effect: *P* = 0.0020) with 1 and 10% *S. fusiforme* supplementation compared to that with 0% supplementation, meaning that *S. fusiforme* inclusion could become detrimental or inhibitory to protozoa numbers (Fig. 3).

### In vitro gas profiles

The dose–response of gas emission profiles of *S. fusiforme* during 72 h of incubation are shown in Table 3. These profiles showed that there were significant increase or decrease on total gas (treatment effect: *P* < 0.0001, treatment effect: *P* = 0.0003), methane (linear effect: *P* < 0.0001, treatment effect: *P* = 0.0003) and carbon dioxide (treatment effect: *P* < 0.0001, treatment effect: *P* < 0.0001) emissions at 3 and 24 h of incubation with *S. fusiforme* at 1 and 10% dose levels compared to those with 0% inclusion of *S. fusiforme*. However, we found that the reduction in gas emission by *S. fusiforme* was time-and-dose dependent, because gas emission was minimal during the first 3 and 24 h but rapidly increased after 48 h incubation.

**Table 3:** Dose response of *Sargassum fusiforme* on in vitro gas profile.

Parameters	*Sargassum fusiforme*					SEM	*P* value		
	0%	1%	3%	5%	10%		*T*	*L*	*Q*
**Total gas emission (mL g**^**−1**^** DM)**									
3 h	153.97^a^	149.38^c^	154.70^a^	144.39^d^	151.50^b^	0.48	< 0.0001	0.0016	< 0.0001
6 h	163.02	168.25	163.82	160.97	160.86	3.88	0.6702	0.3348	0.8848
9 h	172.64	182.46	171.26	167.09	172.58	5.34	0.4009	0.4482	0.3684
12 h	174.27	177.60	182.14	186.73	178.45	5.53	0.5841	0.5775	0.1407
24 h	223.02^a^	195.65^b^	218.78^a^	209.08^ab^	203.57^b^	3.42	0.0003	0.0516	0.8033
48 h	232.09^ab^	238.22^ab^	240.44^a^	234.26^ab^	225.33^b^	3.89	0.1327	0.0679	0.1001
72 h	248.83	247.67	248.30	244.71	254.16	3.49	0.4681	0.2770	0.1930
**Methane emission (mL g**^**−1**^** DM)**									
3 h	1.70^a^	1.17^b^	1.59^a^	1.03^b^	0.92^b^	0.07	< 0.0001	< 0.0001	0.3310
6 h	1.89	2.22	1.86	1.90	1.98	0.31	0.9204	0.8983	0.8100
9 h	2.56	3.30	2.58	2.31	2.94	0.43	0.5499	0.9886	0.3912
12 h	3.30	3.47	4.20	4.61	3.88	0.55	0.4751	0.3842	0.1234
24 h	8.38^a^	4.86^c^	7.75^ab^	6.81^ab^	5.92^bc^	0.44	0.0003	0.0682	0.6578
48 h	9.96	11.16	12.89	10.77	10.86	1.35	0.6443	0.9397	0.3829
72 h	13.58	13.17	15.63	13.27	14.22	0.89	0.3401	0.6701	0.6243
**Carbon dioxide emission (mL g**^**−1**^** DM)**									
3 h	15.03^a^	11.55^bc^	13.69^ab^	9.67^ cd^	8.44^d^	0.55	< 0.0001	< 0.0001	0.2310
6 h	14.68	16.94	14.82	15.21	15.81	1.67	0.8692	0.9290	0.8413
9 h	18.96	21.86	18.98	17.24	20.82	2.16	0.6133	0.9393	0.3440
12 h	24.41	22.63	23.92	25.78	22.55	1.92	0.7426	0.7721	0.4211
24 h	38.88^a^	28.68^c^	35.79^ab^	33.11^bc^	29.37^c^	1.13	< 0.0001	0.0015	0.8011
48 h	43.07	45.14	50.22	44.21	48.21	4.08	0.7187	0.5167	0.7508
72 h	52.19^ab^	49.75^b^	60.25^a^	50.27^b^	52.21^ab^	2.48	0.0772	0.9288	0.3372

SEM, standard error of the mean, n = 3; DM, dry matter; T, treatment; L, linear; Q, quadratic.

^a,b,c,d^Means in a row followed by different superscript letters are significantly different (*P* < 0.05).

### In situ dry matter disappearance (DMD)

The results of in situ DMD are shown in Table 4. A wide range of DMD values showed significant differences (*P* < 0.0001) between timothy hay and *S. fusiforme* in the early to mid-digestion period in terms of their chemical compositions and cell wall constituents. The rapidly soluble fraction ‘a’ of *S. fusiforme* 443.02 g∙kg^−1^ DM was much higher than that of timothy hay 128.58 g∙kg^−1^ DM. In contrast, the insoluble potentially degradable ‘b’ fraction, which is an indicator of rumen microbial degradation, contained much less *S. fusiforme* 323.41 g∙kg^−1^ DM than timothy hay 640.64 g∙kg^−1^ DM. The ‘c’ fraction and effective rumen degradability (ERD) values of *S. fusiforme* 1.12 g∙kg^−1^ DM per hour and 513.80 g∙kg^−1^ DM, respectively and timothy hay 2.14 g∙kg^−1^ DM per hour and 454.70 g∙kg^−1^ DM, respectively were significantly different at a ruminal passage rate of 40 g∙kg^−1^ DM per hour.

**Table 4:** In situ dry matter disappearance and degradation parameters of the basal diet and *Sargassum fusiforme.*

Parameters	Dose		SEM	*P* value	Mean
	Timothy hay	*Sargassum fusiforme*			
**Disappearance of dry matter (g∙kg**^**−**^^**1**^** DM)**					
6 h	219.3^b^	453.7^a^	13.12	0.0002	33.65
12 h	267.1^b^	494.6^a^	8.65	< 0.0001	38.08
24 h	359.3^b^	526.1^a^	3.68	< 0.0001	44.27
48 h	577.3	566.0	4.32	0.1373	57.17
72 h	616.0	627.3	3.72	0.0991	62.16
**Degradation parameters of dry matter**^**1**^** (g∙kg**^**−**^^**1**^** DM)**					
a	128.58	443.02			
b	640.64	323.41			
c	2.14	1.12			
**Effective rumen degradability**^**2**^** (g∙kg**^**−**^^**1**^** DM)h**^**−**^^**1**^					
p = 40	454.70	513.80			

SEM, standard error of the mean; n = 3.

^1^Parameter were calculated by P = a + b(1-e^−ct^). P = dry matter disappearance at time (g∙kg^−1^ DM); a = rapidly soluble fraction (g∙kg^−1^ DM); b = insoluble potentially degradable fraction (g∙kg^−1^ DM); c = constant rate of disappearance for b fraction (g∙kg^−1^ DM)h^−1^; and t = fermentation h in the rumen.

^2^Values were calculated by ERD = a + b(c/(c + p)). p = ruminal passage rate 40(g∙kg^−1^ DM)h^−1^.

^a,b^Means in a row followed by different superscript letters are significantly different (*P* < 0.0001).

## ^1^H nuclear magnetic resonance (^1^H-NMR) profile of ***S. fusiforme***

A representative complete list of ^1^H -NMR metabolite concentrations obtained from *S. fusiforme* is shown in (Table 5). All metabolites could be categorized into seven chemical classes. The classes with the most metabolites were carbohydrates (7), amino acids (6), lipids (3), organic compounds (3), organic acids (5), carboxylic acids (4), and amines (3). Among the metabolites quantified, mannitol 25.84 ± 1.57 mM, guanidoacetate 14.12 ± 1.03 mM and ethylene glycol 2.75 ± 0.24 mM had the highest concentration. All concentrations were representative of the means of triplicate values.

**Table 5:** Identified metabolite concentrations in *Sargassum fusiforme* by ^1^H-NMR (μM) analysis.

Metabolite	Concentration
**Carbohydrates**	
Mannitol (mM)	25.84 ± 1.57
Isocitrate	394.53 ± 6.90
Threonate	351.57 ± 16.14
Glucose-6-phosphate	119.06 ± 7.42
Galactonate	101.27 ± 7.91
Xylose	56.13 ± 5.93
Glucose	29.37 ± 2.84
**Amino acids**	
Alanine (mM)	2.20 ± 0.15
Aspartate	633.57 ± 49.66
Glycine	112.60 ± 14.61
Valine	68.53 ± 6.14
Methionine	49.47 ± 6.70
Leucine	44.57 ± 4.11
**Lipids**	
Ethylene glycol (mM)	2.75 ± 0.24
2-Methylglutarate	120.27 ± 20.94
Valerate	53.75 ± 4.31
**Organic compounds**	
Allantoin	136.90 ± 28.04
Carnitine	94.07 ± 8.25
Caffeine	5.87 ± 0.70
**Organic acids**	
Taurine	130.77 ± 10.38
2-Hydorxyisobutyrate	51.17 ± 7.31
Glycolate	48.50 ± 38.76
3-Hydroxybutyrate	44.10 ± 5.09
Acetoacetate	16.05 ± 1.77
**Carboxylic acids**	
Guanidoacetate (mM)	14.12 ± 1.03
Formate	92.77 ± 6.64
Maleate	7.33 ± 1.22
Fumarate	2.27 ± 0.15
**Amines**	
N-Nitrosodimethylamine	177.87 ± 10.11
Dimethylamine	148.15 ± 30.19
Methylamine	4.00 ± 2.12

All values represent the mean of triplicates ± standard deviation.

## Discussion

*S. fusiforme* is substantially different from terrestrial plants with regard to its chemical composition, cell wall constituents, and morphological characteristics. In particular, the chemical composition of algae varies with the growth environment and season^4^. From a nutritional point of view, they can be dried and used as an ingredient in the total mixed ration (TMR) of ruminants. A TMR is an effective way to supply nutrients to ruminants throughout the day by promoting healthy rumen function.

The high concentration of minerals in *S. fusiforme* might be able to provide a natural source of ruminant feed ingredients Fig. 1. Calcium (Ca^2+^) and magnesium (Mg), in particular, are co-factors for many enzymes and are involved in energy metabolism, protein synthesis, the proper functioning of skeletal muscles, and bone growth^11^. An abundance of essential trace minerals, such as iron (Fe), zinc (Zn), manganese (Mn), and copper (Cu), are also important for the maintenance of normal physiological functions in the body^12^. Trace minerals help to regulate the activities of biochemical processes and compounds in the body, such as transcription factors, growth factors, enzymes, and molecules that are important for the immune response, but are also needed for optimum health and productivity^13,14^. According to one study^15^, *S. fusiforme* contains iodine (average, 151.5 ± 1.03 mg∙kg^−1^ as-raw) and the amount of iodine it contains falls within a range that makes it safe to consume without causing health issues in ruminants^16^. The high salinity content of *S. fusiforme* might induce problems in the rumen microbial ecosystem, which is why its salinity should be reduced by washing *S. fusiforme* prior to using it in the TMR. With regard to salinity, certain in vivo results have showed that cows that consume 0.82 kg of salt daily do not exhibit altered cellulose digestibility and gross energy efficiency compared to those in animals that ate the same diet without salt^17^. In addition, another study^18^ also reported that salt has no detrimental effects on nitrogen balance, rumen fermentation, methane emissions, and energy efficiency.

Due attention must be given to the heavy metal concentrations of *S. fusiforme*, as heavy metal accumulation in ruminants would have adverse effects on health. Brown algae have higher arsenic concentrations than red or green algae, and *S. fusiforme* is known for its high concentration of arsenic^19,20^. However, most algae-derived arsenics are combined with organic molecules and are collectively known as arsenosugars; however, these are not as toxic as their inorganic forms^21^. According to Beresford et al.^22^, the true absorption coefficient of ruminants is considerably lower than that of non-ruminant animals that demonstrate the complete absorption of inorganic arsenic. In addition, the Japanese Ministry has stated that *S. fusiforme* is a good source of dietary fiber and essential minerals for humans^23^. Nevertheless, heavy metal accumulation could be harmful for both animals and humans, through ingestion, which is why the European Commission stated that more research is needed in this area.

In the present study, *S. fusiforme* was found to contain high proportions of water-soluble carbohydrates such as starch and NFC, which are expected to be rapidly fermented by rumen microbes and result in low rumen pH values. The current study revealed improved DM digestibility and microbial growth rates in the early fermentation period, which could be related to the ability of the NFC and minerals in *S. fusiforme* to improve energy availability in microbes in the rumen, because soluble carbohydrate degradation is related to microbial growth and ruminal pH^24^. Maintaining the great diversity of microbes in the rumen is at the core of growth performance and productivity in ruminants, and *S. fusiforme* might provide suitable carbon sources for microbes. As the end products of rumen microbial fermentation, VFAs promote the structural development of the rumen epithelium and preserve healthy microbiota. According to Pié et al.^25^, diet supplementation with prebiotics, such as mannitol in *S. fusiforme*, influences VFA production, the proportions of branched chain amino acids, and ammonia in the gut. Prebiotics, which include oligosaccharides, are a non-digestible food ingredient that stimulate the activities of a number of microbes in the gut^26^.

In the present study, there was an obvious connection between *S. fusiforme* supplementation and VFA production (Fig. 2). Higher proportions of propionate might contribute to reduced energy loss in the form of methane because propionate production occurs separately from the hydrogen production required for methane formation^27^. Based on correlation coefficients, methane was positively correlated with total VFA concentrations (*R*^2^ = 0.6406, *P* < 0.001) and propionate concentrations (*R*^2^ = 0.6087, *P* < 0.001). These results highlight the potential of the functional compounds of *S. fusiforme* to be used as feed ingredients, which could increase VFA production.

Combining the results of total gas and methane production, an ideal supplement should suppress methanogenesis without impairing total gas production, since the latter is positively correlated with the digestibility of feedstuff^28^. Similarly, in our study, supplementation with 1 and 10% *S. fusiforme* was found to suppress methanogenesis at 3 and 24 h, but also simultaneously decreased DM digestibility and total gas production. We hypothesized that several reasons contribute to the aforementioned differences, due to its in vitro incubation in a sealed system. First, during in vitro incubation gas is produced in two ways, direct gas from fermentation and indirect gas produced via the reaction of VFA with bicarbonates, which is an acid–base reaction that maintains pH in the solution^29^. Seo et al.^30^ reported that nitrogen-containing compounds, such as crude proteins, can inhibit this reaction by binding to hydrogen ions and increasing the pH of the solution. Likewise, the amount of gas production might decrease and VFA concentrations and pH could increase in association with a *S. fusiforme* dose–response at 24 h of incubation. Second, brown algae have developed defensive strategies to survive in severe environments, and produce a broad range of chemical compounds known as phlorotannins^31^. Phlorotannin-producing brown algae might contain polar, non-phenolic metabolites that serve as chemical defense mechanisms, preventing them from being consumed by herbivores^32^ and likely altering rumen microbial communities, depending on the substrates consumed^33^. In fact, the current results indicate that numbers of protozoa (which is related to rumen methanogenesis) decreased after 24 h of incubation. This might be particularly relevant in this context because Belanche et al.^34^ also reported that phlorotannins have potential anti-protozoal properties and reduce total gas production. Finally, although the compositions of fatty acids (FAs) in *S. fusiforme* were not examined in the present study, it is possible that these FAs can mitigate methane emissions via direct toxic effects on rumen microbes, including methanogens^35^. According to one study^36^, palmitic acid (C_16:0_) is the most common type of FA in *S. fusiforme*, and its derivative hexadecatrienoic acid (HA, cis-C_16:6,9,12_) has the potential to suppress methane emissions^37^. Presumably, this might be mediated in the rumen via extensive biohydrogenation of the HA in *S. fusiforme*, which resulted in lower methane emissions at 3 and 24 h of incubation with a high dose of *S. fusiforme*. However, the actual methane emissions for *S. fusiforme* were lower than the theoretical methane emission values estimated based on VFA proportions with 1 and 10% *S. fusiforme* supplementation at 24 h incubation^38^*.* Inconsistencies with experimental methane emissions might be related to a simultaneous increase in acetate and butyrate concentrations based on result of the current study. In general, ruminal methane formation is positively correlated with an increase in acetate and butyrate, and in fact, the degradation of mannitol present in *S. fusiforme* can lead to increased acetate and butyrate concentration during anaerobe fermentation^39^.

However, the reasons for the lack of a significant effect on methane and carbon dioxide emissions at 48 and 72 h are still unclear, although their levels increased partly because of changes in ruminal fermentation and microbial populations. According to a previous study^40^, bromoform, which is a type of halogen compound found in the red algae genus *Asparagopsis*, effectively reduces methane emissions based on an in vitro assessment, but these emissions vary depending on the type of substrate consumed. This suggests that *S. fusiforme,* which contains phlorotannins, can also affect methane emissions if the rumen microbial communities in question have been altered using different substrates. Moreover, Milledge et al.^41^ reported that arsenic present in *Sargassum* species has a strong inhibitory effect on methane potential during anaerobic digestion, depending on its type. Therefore, further work on speciation with respect to arsenic in *S. fusiforme* is required to fully determine the relationship between their form and methane potential, as well as the risk to health and agricultural environment.

Profiling in situ DMD and degradation parameters is important to evaluate the nutritional value of ruminant feed. Assessing degradation parameters comprises methods to determine the amount of nutrients digested in sections of the digestive tract and how these nutrients can be absorbed and be available to ruminants^42^. This can offer insights into the proper feeding and management of ruminants. The rapidly soluble fraction ‘a’ varied considerably between timothy hay and *S. fusiforme*. The reason for this is that the latter has a much higher content of crude ash and a high proportion of NFC in its cell walls, which are promptly digested in the rumen fluid. In agreement with our results, Tayyab et al.^4^ reported a higher rate of degradation in the ‘a’ fraction of algae. The insoluble potentially degradable ‘b’ fraction was found to be higher in timothy hay than in *S. fusiforme*. Based on the cell wall constituents of *S. fusiforme*, ruminants should be able to digest *S. fusiforme* given that rumen microbes are able to digest even unprocessed algal cell walls. However, the current results can be explained by the large proportion of lignin and polysaccharides in *S. fusiforme* that inhibit cell wall degradation and enzyme digestion by rumen microbes, which are exacerbated by the lack of adaptation time to *S. fusiforme*. Typically, *S. fusiforme* has relatively lower proportion of lignin compared to other brown algae species *Sargassum fulvellum*^1^. As stated previously, the results of this study, indicated that the degradation parameters differed significantly between feedstuffs, and the ‘a’ fraction and ERD values varied considerably with the chemical composition and morphological type of the feedstuff. One reason for the variation in the ‘a’ fraction and ERD values could be the small feed particle size after processing. According to Anele et al.^43^, particles with smaller sizes are often more readily washed out from bags during in situ incubation, and Morris^44^ also reported that hardness could influence the distribution of particle sizes during the milling process. Although the current study did not consider particle size or hardness, the results were consistent with those of Tayyab et al.^4^. Nonetheless, these factors should be the subject of future studies.

Naturally occurring bioavailable compounds and metabolites are considered safe for human health and therefore could also be widely utilized as feed ingredients to provide many functional effects. Most algae contain all essential amino acids, but the concentrations of these amino acids vary greatly from one algal phylum to another, within each phylum, and even from one species to another within the same genus^45^. In agreement with these results, the most abundant dietary amino acids in *S. fusiforme* were alanine, aspartate, and methionine; however, one study^46^ previously found more amino acids such as lysine, arginine, and glutamine. Such fluctuations are believed to be linked to environmental factors such as nutrient supply, pH, seasonal periods and species. Generally, alanine helps to develop ruminant muscles, aspartate acts as an antimicrobial, which can stimulate the growth of rumen microbes^47^, and methionine has been linked to wool production^3^. However, to maximize amino acid utilization in ruminants, diets are typically formulated by mixing different feedstuffs to form a balanced combination of amino acids that meet their nutritional requirements^48^.

A vast number of in vitro studies have demonstrated the potential biological activity of polysaccharides in brown algae with respect to ruminant nutrition^1,49^. Mannitol, a sugar alcohol equivalent to mannose, is one of the primary carbon sources for brown algae. Carbohydrates in brown algae are soluble and available and can be directly utilized by microbes as a carbon source^50^. Although the molecular mode of action of mannitol in ruminants has not been explicitly elucidated, some recent reports have described that mannitol can promote VFA production, the effects of which depend on activation through anaerobic fermentation and conversion to acetate and butyrate^39^. This finding is in agreement with our results, wherein an abundance of mannitol might have contributed to VFA production.

Metabolites in *S. fusiforme*, such as ethylene glycol and guanidoacetate were found in much higher quantities than those of other metabolites. Ethylene glycol has been largely used to alleviate the effects of condensed tannins and polyphenol content, which in turn increases the absorption of amino acids in the rumen, and that consequently increases milk yield and wool production^51^. In contrast, the natural biosynthetic precursor of creatine is guanidoacetate; however, its bioavailability has not yet been investigated in ruminants. According to our previous study^1^, it is associated with muscle energy metabolism and the urea cycle. Increasing the efficiency of the urea cycle in ruminants could contribute to a decrease in urea-nitrogen excretion in the urine, which would minimize the negative effects of nitrogen excretion on the environment^52^. In addition, some rumen methanogens can possibly use methyl-containing compounds such as trimethylamine and dimethylamine to form methane^53^, and *N*-methylate amines might provide an additional source of ammonia to rumen bacteria^54^. Although methanogen distributions in the rumen were not investigated in the present study, the presence of dimethylamine in *S. fusiforme* might allow for the growth of methanogens, which would be expected to affect methane emissions after 48 h incubation. These findings demonstrate the necessity of further studies, based on controlled environmental conditions in the laboratory and in the field. Therefore, *S. fusiforme* seems to be a promising feed ingredient for ruminants as it contains natural forms of minerals and amino acids that are easily bioavailable and can even be manipulated via the process of bio-absorption to increase nutritional benefits.

In conclusion, the brown algae *S. fusiforme* has the potential to be an alternative, renewable feed ingredient for ruminants, not only to improve fermentation characteristics but also to manipulate ruminant gas emissions. *S. fusiforme* contains valuable metabolites and nutrients, which play important roles as prebiotics, and this is expected to replace other supplementary feeds. In addition, this has environmental and economic benefits as it provides an avenue for algal waste management. Further studies are needed to determine the fatty acid compositions in *S. fusiforme* and their effects on rumen microbial communities. Additionally, the efficiency of *S. fusiforme* to reduce methane emissions should be evaluated using different substrates. The findings of this study need to be investigated using an in vivo study to establish their veracity with respect to the animal itself.

## Materials and methods

### Ethics statement

All the experimental protocol of this experiment was approved by the Animal Care and Use Committee (approved ID: GNU-180130-A0007) of Gyeongsang National University (Jinju, Gyeongsangnam-do, Korea). All experiments procedure was carried out according to the guidelines and regulations set out by this governing body.

### Raw materials preparation

The raw material preparation was similar to those of Choi et al.^1,49^. In brief, *S. fusiforme* was collected in March 2018 from Hansan island located in the coastal area of Tongyeong (Gyeongsangnam-do province of Korea). Morphological identification was accomplished using published literature^55^. After collection, *S. fusiforme* was rinsed in fresh water and cleaned of epibionts, sand, and detritus. Subsequently, *S. fusiforme* was dried in a 55 °C hot-air drier overnight and then cut into small pieces. Dried *S. fusiforme* and timothy hay were ground through a 2 mm screen with a Wiley mill (Arthur H. Thomas, Philadelphia, PA) for in situ analysis, and a 1 mm screen for in vitro and chemical analysis.

### Chemical analysis

All the contents of dry matter (DM; Method 934.01), crude protein (CP; Method 954.01), crude fiber (CF; Method 962.09), ether extract (EE; Method 920.39) and crude ash (CA; Method 942.05) in timothy hay and *S. fuisfomre* were assayed as described by AOAC^56^. All the content of neutral detergent fiber (NDF), acid detergent fiber (ADF) and lignin were analyzed according to the procedures of Van soest et al.^57^. Non-fiber carbohydrate (NFC) content was estimated based on the following equation : NFC = 1000-(CP + EE + CA + NDF) and nitrogen free extract (NFE) content was estimated based on the following equation : NFE = 1000-(CP + EE + CA + CF). As stated in Choi et al.^1,49^, concentration of mineral and heavy metal in *S. fusiforme* were determined by AAS (atomic absorption spectrophotometry, Hitachi Z, 2000, Polarized, Zeman, Tokyo, Japan) and ICP OES (inductively coupled plasma optical emission spectrometry) (Perkin-Elmer, Optima 4300 DV, Shelton, CT 06,484–4794, USA). Mercury (Hg) concentration was used for determination by (Perkin-Elmer, FIMS-100, MA, USA). Gross energy (GE) was assayed by combustion in an adiabatic bomb calorimeter (Werke C2000, IKA, Staufen, Germany).

### Ruminal inoculum and in vitro trial

Two rumen fistulated, non-lactating Hanwoo cows body weight (BW) = 450 ± 30 kg, were used as ruminal inoculum donors. Cows were fed a basal diet of 60% timothy hay and 40% commercial concentrate at maintenance energy level (2% DM of their BW) and had free access to clean drinking water and a mineral block. Diets were fed twice daily at 0900 and 1700 h. The rumen fluid from the two donors was mixed and filtered through 4 layers of cheesecloth into a pre-warmed insulated bottle and brought immediately to the laboratory. The rumen fluid was mixed with McDougall’s artificial saliva at a ratio of 1:2 (v/v) and maintained at 39 °C under strictly anaerobic environments.

Timothy hay supplied to cows, was used as basal substrate and *S. fusiforme* was used at 4 dose levels: 1%, 3%, 5%, and 10% of the timothy hay. Approximately, 0.300 mg of timothy hay and each dose levels of *S. fusiforme* were placed into pre-weighed nylon bags (pore size 50 ± 10 μm, R510, Ankom Technology, NY, USA) and transferred in 50 mL serum bottle. Then, the respective mixtures were accurately dispensed 15 mL into serum bottle flushed with O_2_-free N_2_. Tubes were capped with a butyl rubber and sealed with an aluminum cap. Placing in shaking incubator at 120 rpm for 3, 6, 9, 12, 24, 48 and 72 h at 39 °C. After incubating for each time, samples were placed on ice to stop the fermentation. In vitro incubations were conducted in triplicate of samples with a completely randomized design. All the procedure of ruminal inoculum and in vitro trail were proceeded according to those of Choi et al.^1,49^.

### Profiles of gas and rumen fermentation

To measure total gas production on in vitro system, we used method by Theodorou et al^58^ and converted to gas volume (mL) from the equation for our laboratory conditions.

$${\text{V}} = \left( {{\text{P}}{-}{21}.0{16}} \right)/{16}.{132}$$

where V = gas volume (mL); P = measured pressure (psi).

For the gas profiles, headspace gas (5 mL) was collected from serum bottle each incubation using sealed gas injection syringes and transferred into a vacuum test tube (Vacutainer, Becton Dickinson, Franklin Laker, NJ, USA). Gas chromatography (Agilent Technologies HP 5890, Santa Clara, CA, USA) was conducted to analyze for methane and carbon dioxide concentration equipped with a thermal conductivity detector and Carboxen 1006 PLOT capillary column 30 mm × 0.53 mm (Supelco, Bellefonte, PA, USA).

The culture medium was subsampled to assay pH, volatile fatty acid (VFA), and microbial growth rate. The pH was measured with pH meter (MP230, Mettler-Toledo, Greifensee, Switzerland). Cultured medium was centrifuged at 10,483×*g* for 3 min and supernatant was collected for VFA analysis. The concentration of VFA was measured with a high performance liquid chromatography (L-2200, Hitachi, Tokyo, Japan) fitted with a UV detector (L-2400, Hitachi, Tokyo, Japan) and a column (Metacarb 87H, Varian, CA, USA), according to the methods described by Adesogan et al.^59^. To evaluate microbial growth rate, cultured mediums from the centrifugation at 655×*g* for 3 min and then washed with sodium phosphate buffer for four times at 14,269×*g* for 3 min and assayed to optical density (OD) at 550 nm by spectrophotometer (Model 680, Bio-Rad Laboratories, Hercules, CA, USA).

For protozoa count, 2 mL of the rumen fluid after 24 h incubation was mixed with 2 mL of methylgreen-formalin-saline (MFS) solution consisting of 100 mL of 35% formaldehyde solution, 0.6 g of methyl green, and 8.0 g of NaCl and 900 mL distilled water^60^. Protozoa were microscopically enumerated using a counting chamber (Neubauer Improved Bright-Line counting cell, 0.1-mm depth; Hausser Scientific Co., Horsham, PA). Counting of each sample was performed in triplicates, and if the average of the triplicates differed by more than 10% the counts were repeated.

In vitro DM digestibility was estimated using the nylon bag digestion method. Briefly, after incubation, the nylon bag with substrate was washed three times and then oven dried at 105 °C for 24 h. DM digestibility was calculated as equation below. Where DM_I_ means initial DM and DM_F_ means finished DM.

1 $${\text{DM digestibility }}\left( {{\text{g}}\;{\text{kg}}^{ - 1} {\text{ of DM}}} \right) = \frac{{weight\;of\;DM_{I} - weight\;of\;DM_{f} }}{{weight\;of\;DM_{I} }} \times 1000{ }$$

### In situ trial

Dry matter disappearance (DMD) derived from timothy hay and *S. fusiforme* was measured in situ using nylon bags techniques as described by Ørskov et al.^61^. Briefly, air-dry nylon bags (pore size 50 ± 10 μm, R510, Ankom Technology, NY, USA) were filled with 5 g samples of dried timothy hay and *S. fusiforme* that had been ground previously and screened thorough a 2 mm screen. All bags were incubated in the rumen for 6, 12, 24, 48 and 72 h; there were triplicate per each sample. After the incubation was completed, bags were retrieved, washed under flowing water to stop the fermentation and then dried to a constant weight at 65 °C for 48 h. DMD was calculated with initial DM weight and DM of finished fermentation. The rate of DM disappearance were fitted to the exponential equation Ørskov and McDonald^62^ as $\mathrm{P}=\mathrm{a}+\mathrm{b}(1-{e}^{-ct})$. In these equations, P is the proportion of substrate DM which disappeared at time t, t is the incubation time (hours), a is rapidly soluble fraction, b is insoluble fraction, and c is the constant rate of disappearance for b fraction per hours. The nonlinear (NLIN) procedure of SAS 9.4 version (SAS Institute Inc., Cary, NC) using Marquardt’s algorithm while varying a, b, and c. The effective rumen degradability (ERD) must consider a rate of passage coefficient according to the following equation of Ørskov and McDonald^62^ : ERD = $\mathrm{a}+ \frac{bc}{c+p}$ where p is the estimated rate of passage from the rumen. Hypothetical ruminal passage rates of 4%h^−1^ were used for estimation of ERD of timothy hay and *S. fusiforme*.

### NMR spectroscopy and metabolites identification and quantification

Freeze-dried *S. fusiforme* samples were grounded to a fine powder. Five hundred microliters of methanol-*d4* (99.8%), 350 μL of 0.2 M phosphate buffer solution (0.2 M Na_2_HPO_4_, 0.2 M Na_2_HPO_4_ in D_2_O, pH 7.0), and 100 μL of 5 mM TSP (3-trimethylsilyl propionic-2, 2, 3, 3-*d4* acid sodium salt) were added as extraction solvents to 100 ± 0.5 mg of dried powder. Deuterium oxide (D_2_O) was used as the internal lock signal, and TSP was used as an internal standard with a chemical shift (δ) of 0.0 ppm. Extracted samples were sonicated for 20 min and then centrifuged at 12,303×*g* for 10 min at 4 °C. The supernatants (600 μL) were transferred to 5 mm NMR tubes for NMR analysis. The detail procedures described by method of Jung et al.^63^.

^1^H -NMR spectra of *S. fusiforme* were obtained using an SPE-800 MHz NMR-MS hyphenated system (Bruker BioSpin Co., Billerica, MA) at 64 K using a 5 mm Triple-resonance Inverse (TCI) cryoprobe with Z-Gradients (Bruker BioSpin Co, Billerica, MA).

All spectra were calibrated, phase-adjusted and baseline-corrected by Chenomx NMR Suite 8.4 (Chenomx, Edmonton, AB, Canada), in order to estimate concentration for those metabolites, that were determined by Chenomx NMR Suite profiler.

### Statistical analysis

All statistical analyses were conducted using a one-way analysis of variance (ANOVA) model, with the general linear model procedure of SAS for a completely randomized design. Tukey’s multiple range test was employed for multiple comparisons. Linear and quadratic trends for the samples comparing the dose levels of *S. fusiforme* and the dependent variables were tested using polynomial contrasts. Unequally spaced doses of *S. fusiforme* were obtained using the interactive matrix language procedure of SAS. Results were considered statistically significant at a value of *P* < 0.05. The relationship between methane and VFA concentration was fitted using the Pearson product moment correlation coefficient. Regression assumptions were checked using PRESS (predicted residual error sum of squares), the Durbin-Watson Statistic, Kolmogorov–Smirnov Statistic, and Constant Variance Test in this program. Sigmaplot (version 10.0 for Windows, Sigmaplot, Corvallis, OR, USA) was used for this analysis.

## Abbreviations

ADF: Acid detergent fiber
BW: Body weight
CA: Crude ash
CF: Crude fiber
CP: Crude protein
DM: Dry matter
DMD: Dry matter disappearance
EE: Ether extract
ERD: Effective ruminal degradability
FAs: Fatty acids
^1^H-NMR: ^1^H nuclear magnetic resonance
NDF: Neutral detergent fiber
NFC: Non-fiber carbohydrate
OD: Optical density
TMR: Total mixed ration
*S. fusiforme*: *Sargassum fusiforme*
VFA: Volatile fatty acid
